# Twice-daily versus once-daily lisinopril and losartan for hypertension: Real-world effectiveness and safety

**DOI:** 10.1371/journal.pone.0243371

**Published:** 2020-12-03

**Authors:** Catherine G. Derington, Jordan B. King, Thomas Delate, Sheila R. Botts, Miranda Kroehl, David P. Kao, Katy E. Trinkley

**Affiliations:** 1 Department of Population Health Sciences, School of Medicine, University of Utah, Salt Lake City, UT, United States of America; 2 Institute for Health Research, Kaiser Permanente Colorado, Aurora, CO, United States of America; 3 University of Colorado Skaggs School of Pharmacy and Pharmaceutical Sciences, Aurora, CO, United States of America; 4 Drug Use Management, Kaiser Permanente National Pharmacy, Aurora, CO, United States of America; 5 Department of Pharmacy, Kaiser Permanente Colorado, Aurora, CO, United States of America; 6 Colorado School of Public Health, Aurora, CO, United States of America; 7 Cardiac and Vascular Center, University of Colorado Health, Aurora, CO, United States of America; 8 School of Medicine, University of Colorado, Aurora, CO, United States of America; International University of Health and Welfare, School of Medicine, JAPAN

## Abstract

**Background:**

Lisinopril and losartan manufacturer labels recommend twice-daily dosing (BID) if once-daily (QDay) is insufficient to lower blood pressure (BP).

**Methods and results:**

Retrospective cohort study of patients taking QDay lisinopril and losartan who experienced a dose-doubling (index date). A text-processing tool categorized BID and QDay groups at the index date based on administration instructions. We excluded: pregnant/hospice, regimens other than BID/QDay, and without BP measurements -6 months/+12 months of the index date. The most proximal BP measurements -6 months and +2 weeks to 12 months of the index date were used to evaluate BP differences. Propensity scores were generated, and differences in BP and adverse events (angioedema, acute kidney injury, hyperkalemia) between BID/QDay groups were analyzed within dosing cohorts using inverse propensity of treatment-weighted regression models. Of 11,210 and 6,051 patients who met all criteria for lisinopril and losartan, 784 (7.0%) and 453 (7.5%) were taking BID, respectively. BID patients were older and had higher comorbidity and medication burdens. There were no differences in systolic/diastolic BP between BID and QDay, with absolute differences in mean systolic BP ranging from -1.8 to 0.7 mmHg and diastolic BP ranging from -1.1 to 0.1 mmHg (all 95% confidence intervals [CI] cross 0). Lisinopril 10mg BID was associated with an increased odds of angioedema compared to lisinopril 20mg QDay (odds ratio 2.27, 95%CI 1.13–4.58).

**Conclusions:**

Adjusted models do not support improved effectiveness or safety of BID lisinopril and losartan.

## Introduction

High blood pressure (BP) remains the leading modifiable risk factor for cardiovascular morbidity and mortality globally [1, 2], even though effective and safe antihypertensive medications are generic, widely available, and low-cost. Lisinopril and losartan, the most common angiotensin converting enzyme inhibitor (ACEI) and angiotensin receptor blocker (ARB), respectively [3], have been traditionally dosed once-daily. However, the respective half-lives of lisinopril and losartan are twelve and nine hours, indicating that twice-daily dosing may be more effective than once-daily if the latter does not achieve optimal BP lowering, as suggested by the manufacturer labels [4, 5]. Challenging traditional antihypertensive dosing is not unprecedented; for example, atenolol dosed once-daily has demonstrated poor 24-hour BP reduction and postulated to be why atenolol has not manifested improvements in cardiovascular disease events in randomized clinical trials and meta-analyses [6–8].

We previously reported that lisinopril 20mg twice-daily is more effective than 40mg once-daily, with an average 10.2 mmHg difference in systolic BP (SBP) between the groups [9]. If effective on a population level, this simple intervention could improve BP control and prevent progression of hypertension-related complications. Therefore, we sought to expand on our previous work by using a larger sample size from a different health system, including losartan as an exposure, and evaluating multiple dosing cohorts. We hypothesized that twice-daily dosing would be more effective and equally safe as once-daily dosing.

## Methods

### Study design and setting

This retrospective cohort study was conducted using administrative and claims databases at a large integrated health system, Kaiser Permanente Colorado (KPCO). KPCO utilizes an electronic health record (EHR) to document and store health information. Data were obtained from the KPCO Virtual Data Warehouse (VDW), a data repository used for clinical research containing structured and unstructured data from within and external to the health system [10]. The VDW stores longitudinal data, including *International Classification of Diseases*, *Ninth Edition* (ICD-9) and *International Classification of Diseases*, *Tenth Edition* (ICD-10) diagnoses; procedure and utilization data captured with Current Procedural Terminology codes and Diagnosis-Related Groups; pharmacy dispenses captured with Generic Product Identifier codes and National Drug Codes; patient demographics, vitals, and laboratory measurements; geographic socioeconomic data as defined by the US Census; plan enrollment and membership; and incidences and causes of death.

Internal protocols guided hypertension management according to evidence-based guidelines of the time (i.e., the Seventh and Eighth Reports of the Joint National Committee) and formulary preferences (e.g., generic use), but the protocols did not suggest that clinicians prescribe twice-daily lisinopril or losartan. All aspects of this study were reviewed and approved by the KPCO Institutional Review Board and the Colorado Multiple Institutional Review Board.

### Patient population

**Fig 1:**
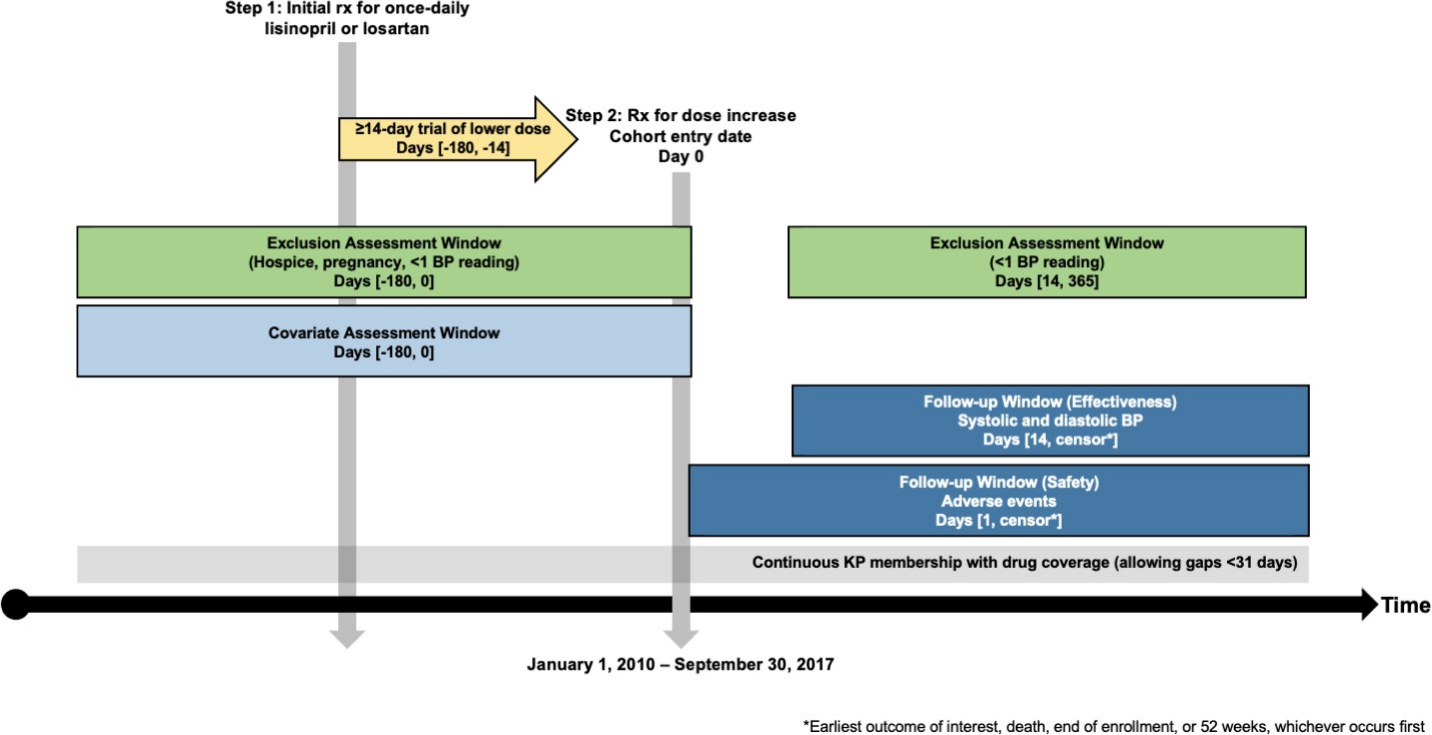
Study design. *Earliest outcome of interest, death, end of enrollment, or 52 weeks, whichever occurs first. BP–blood pressure; KP–Kaiser Permanente.

The study population included KPCO members with hypertension treated with lisinopril or losartan (**Fig 1**). Eligible patients had an initial prescription dispensed for once-daily lisinopril or losartan between January 1, 2010, and September 30, 2017. A subsequent prescription dispense for a doubling of the total daily dose was identified after the initial prescription. The index date was the date of the dose doubling. To be included in the study, the initial prescription had to be dispensed two weeks or more before the index date (i.e., patients were required to have at least two weeks of medication use before they escalated their dose). Two lisinopril dosing cohorts were assessed: a dose increase from 10mg daily to 20mg daily (20mg cohort), and a dose increase from 20mg daily to 40mg daily (40mg cohort). Similarly, two losartan dosing cohorts were assessed: 25mg daily to 50mg daily (50mg cohort) and 50mg daily to 100mg daily (100mg cohort). Any regimens that did not align with these dosing cohorts were excluded (e.g., lisinopril increase from 20mg daily to 30mg daily). Patients could be included in two dosing cohorts if they experienced two eligible dose-doublings (e.g., lisinopril 10mg to 20mg, then 20mg to 40mg); therefore, the cohorts were not mutually-exclusive.

Patients also had to have at least one outpatient BP measurement in the six months before, or on the day of, the index date and at least one outpatient BP measurement two to 52 weeks after the index date to be included (i.e., the variable assessment period). We also excluded patients: 1) without prescription drug coverage; 2) with lapses in membership >30 days; 3) were <18 years of age at the index date; 4) became pregnant, or 5) entered hospice at any time within the variable assessment period.

### Assessment of daily dosing frequency

Within the dosing cohorts, patients were categorized into once-daily or twice-daily dosing groups using administration instructions extracted from the prescription *signatura* (“the sig”) stored in dispensing records. Because the sig contains unstructured free-text data [11–14], a text-processing tool was developed to extract the daily dosing frequency from the sig [15]. Briefly, the tool first standardizes all numbers, fractions, and characters (e.g., “two” was converted to “2”, the ampersand symbol [&] was converted to “and”). Then, the tool standardizes all abbreviations and phrases (e.g., “QAM” is converted to “every morning,” and “evening,” “afternoon,” “night,” “nighttime,” and “bedtime” were converted to “every evening”). Spelling errors are also corrected at this step (e.g., “daly” and “dialy” were converted to “daily”). Next, the tool assigns a value of “1” for each unique daily administration within the sig. The daily dosing frequency is derived from tallying the number of unique daily administrations. The tool has a 99.9% positive predictive value to interpret the sig and assign the correct dosing frequency. Sigs that were not assigned a dosing frequency by the tool were manually reviewed and assigned the correct dosing frequency. Patients with any dosing frequency except once-daily or twice-daily were excluded from the analysis.

### Outcomes

The primary outcome was the follow-up SBP assessed at least two weeks and up to 52 weeks after the index date. Secondary outcomes included diastolic BP (DBP) measured at the same time as the follow-up SBP and adverse drug events (ADEs) that occurred up to 52 weeks after the index date. Within dosing cohorts, the mean absolute differences in SBP and DBP between the once-daily and twice-daily groups were assessed, and patients were categorized as achieving SBP <130 mmHg, 130–139 mmHg, 140–149 mmHg, or ≥150 mmHg, and DBP <80 mmHg, 80–89 mmHg, 90–99 mmHg, or ≥100 mmHg. The most proximal SBP and DBP measurements in outpatient settings relative to the index date were used for analyses. If more than one measurement was taken on the same date, the average was used.

ADEs assessed included hyperkalemia, angioedema, and acute kidney injury (AKI). A laboratory value for serum potassium ≥5.2 mEq/L defined hyperkalemia. Encounter of any type (e.g., hospitalization, emergency room) with an ICD-9 code of 995.1, 995.2, or E942.6 or an ICD-10 code of T46.4X5 or T78.3XXA identified angioedema episodes. Encounters of any type with an ICD-9 code of 584.x, 586.x, 788.5 or an ICD-10 code of N17.x, N19.x, R34.x identified AKI episodes [16].

### Covariates

Age, sex, race-ethnicity, education level, area-based (US Census) median family household income, tobacco use, comorbidities, body mass index (BMI), SBP, DBP, serum creatinine, estimated glomerular filtration rate (eGFR), serum potassium, albuminuria, number of BP measurements, and other BP-affecting medications that were dispensed and the patient had on-hand at the time of the index date were collected from the VDW in the 6 months prior to the index date. A medication “on hand” was defined as a prescription that was dispensed before the index date with a quantity that met or exceeded the index date. Additional medications collected that affect BP included anti-anginals, beta-blockers, calcium channel blockers, diuretics, glucocorticosteroids, immunosuppressants, non-steroidal anti-inflammatory drugs, and selective norepinephrine reuptake inhibitors. Prescribing provider specialty was also included in the calculation of the propensity score, categorized as cardiology, family medicine, internal medicine, nephrology, nurse practitioner, pediatrics, urgent care, other, and missing. However, there was a high level (45–65% depending on the dosing cohort) for this variable, and exclusion of this variable from the model did not substantively affect results, so it was left in the model. Comorbidities were collected from ICD-9 and ICD-10 codes attached to any type of encounter in the six months prior to the index date, which were used to calculate a baseline Charlson Comorbidity Index (CCI) score for each patient [17].

### Statistical analyses

Analyses for all study outcomes occurred within dosing cohorts for each medication and were adjusted for covariates that were clinically and statistically associated with the receipt of twice-daily dosing using propensity scores (i.e., the conditional probability of receiving twice-daily dosing, given the covariates) [18, 19]. Covariates were selected for inclusion in the propensity score based on differences between the daily dosing groups of p<0.2, prior studies [9], and clinical judgement. Covariate balance was assessed by evaluating patient characteristics, before and after weighting, with standardized differences <0.1 considered to be non-clinically meaningful [20]. Standardized differences after weighting for all baseline characteristics were <0.1 before trimming weights and conducting analyses. The propensity score was estimated using a multivariable logistic regression model to predict the probability of receiving twice-daily dosing based on gender, age, race, ethnicity, tobacco use, BMI, number of BP measurements before the index date, CCI, chronic pulmonary disease, congestive heart failure, depression, diabetes, chronic kidney disease, eGFR, albuminuria, other medications that affect BP, and provider specialty. The propensity score was used to construct stabilized inverse probability of treatment weights (IPTW) with trimming for extreme weights <1^st^ percentile and >99^th^ percentile. An analysis utilizing IPTW with stabilized weights was selected over other propensity score methods (e.g., matching) because IPTW most effectively minimizes variance with time-dependent covariates to estimate risk differences while minimizing type I error [21, 22].

Patient characteristics were reported as means ± standard deviations (SD) for normally-distributed data and medians [interquartile ranges, IQR] for non-normally-distributed data, with appropriate tests used to assess for significant differences between the groups (e.g., chi-square, t-test, Wilcoxan rank-sum test). To evaluate the association between daily dosing frequency and BP, IPT-weighted linear regression models were constructed with the follow-up BP as the dependent variable, dosing frequency group as the independent variable, and baseline BP as a covariate. Odds ratios (OR) and 95% confidence intervals (CI) [23] were generated for dichotomous outcomes using IPT-weighted logistic regression models with daily dosing frequency group as the independent variable. To determine if the outcomes were sensitive to the implementation of IPTW, we also analyzed BP outcomes using unadjusted and multi-variable adjusted models.

Data were analyzed using SAS v.9.4 (SAS Institute, Cary, NC).

## Results

### Study population

**Fig 2:**
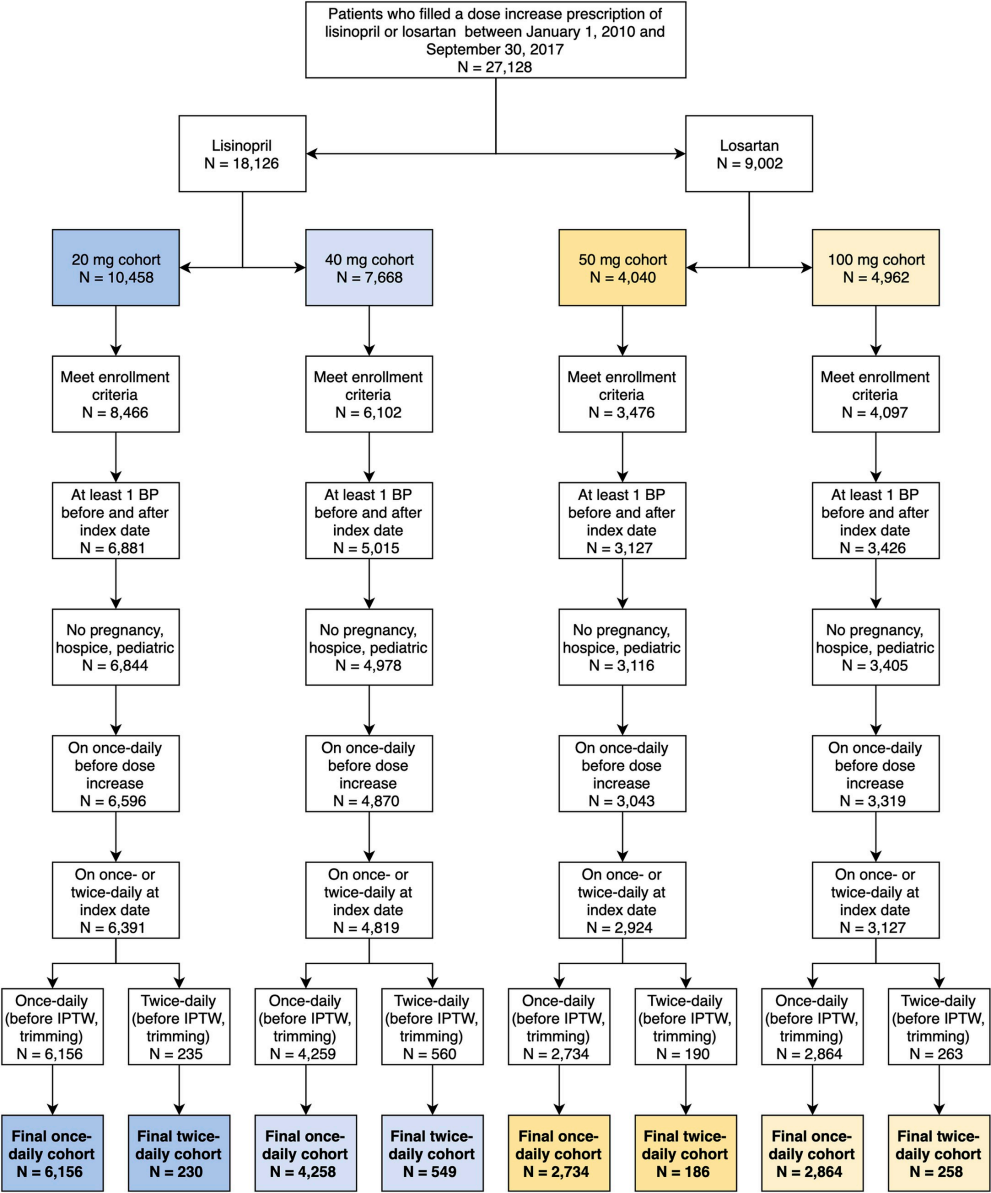
Flowchart showing the application of exclusion criteria, text-processing tool, and IPTW to derive the final analytic cohorts. IPTW–inverse probability of treatment weighting; KPCO–Kaiser Permanente Colorado.

During the study period, 18,126 and 9,002 patients filled a prescription for a dose increase of lisinopril and losartan, respectively (**Fig 2**). After application of all exclusion criteria and trimming for extreme weights, the lisinopril 20mg and 40mg cohorts consisted of 6,386 patients (once-daily n = 6,156, twice-daily n = 230) and 4,807 patients (once-daily n = 4,258, twice-daily n = 549), respectively. The losartan 50mg and 100mg cohorts consisted of 2,920 patients (once-daily n = 2,734, twice-daily n = 186) and 3,122 patients (once-daily n = 2,864, twice-daily n = 258), respectively.

Before applying IPTW, participants who received twice-daily dosing were more likely to be older, Non-Hispanic White patients with lower BMI, higher CCI score, and have chronic pulmonary disease, congestive heart failure, depression, and chronic kidney disease (**S1 and S2 Tables**). Patients taking twice-daily dosing also had lower SBP, DBP, and eGFR, and were more likely to have other medications on-hand that affect BP.

### Blood pressure outcomes

#### Lisinopril

**Fig 3:**
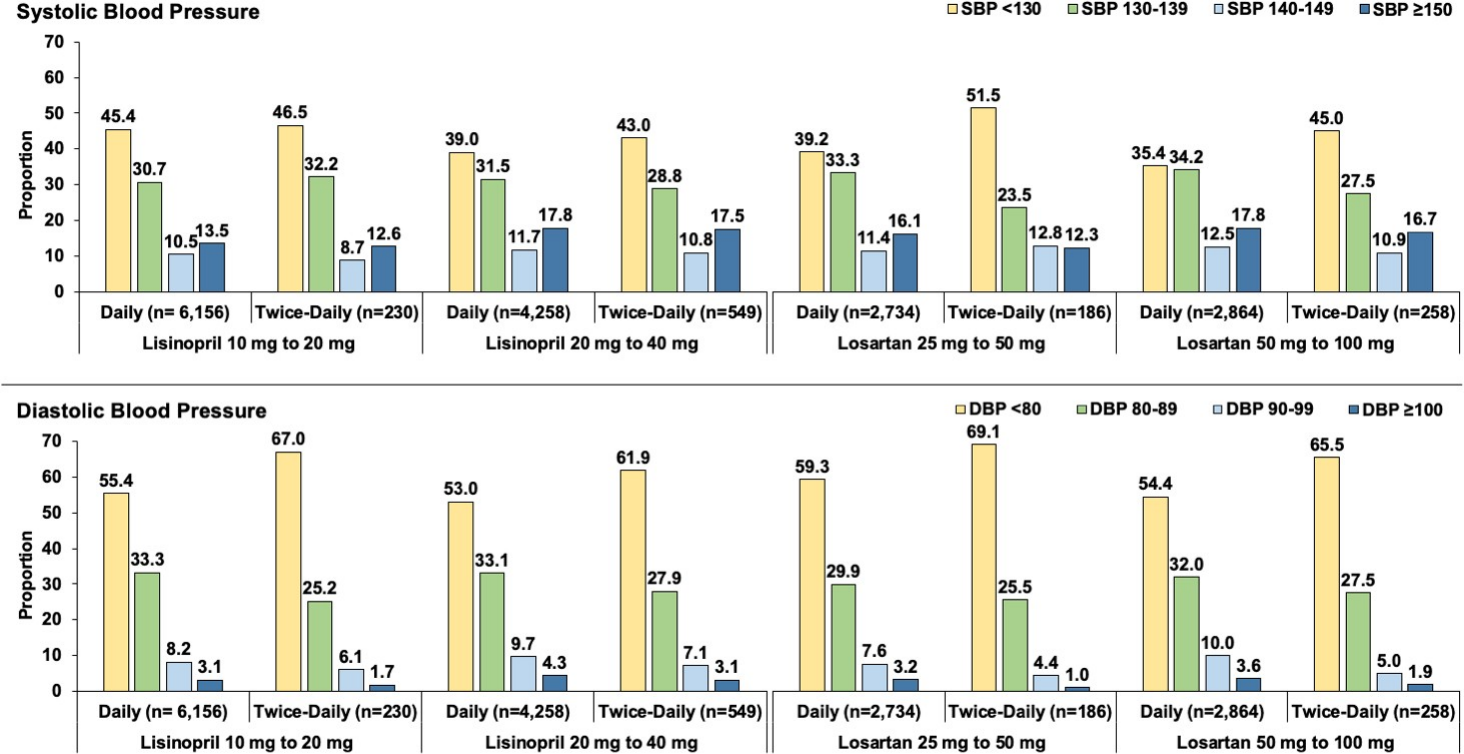
Achieved systolic (Panel A) and diastolic blood pressure (Panel B) among patients taking twice-daily versus once-daily lisinopril and losartan. Panel A: Proportion of patients achieving SBP <130 mmHg, 130–139 mmHg, 140–149 mmHg, and ≥150 mmHg. Panel B: Proportion of patients achieving DBP <80 mmHg, 80–89 mmHg, 90–99 mmHg, and ≥100 mmHg. SBP–systolic blood pressure; DBP–diastolic blood pressure.

In the lisinopril 20mg cohort, the average number of BP measurements in the 6 months prior to the index date was 3.6±2.9 and 4.7±3.3 in the once-daily and twice-daily groups, respectively, and the median time from index date to follow-up BP measurement was 76 days [IQR 35,171] and 72 days [IQR 42,151] for the once-daily and twice-daily groups, respectively. For the lisinopril 40mg cohort, the average number of BP measurements in the 6 months prior to the index date was 3.6±2.9 and 4.3±3.5 in the once-daily and twice-daily groups, respectively, and the median time from index date to follow-up BP measurement was 76 days [IQR 25,172] and 78 days [IQR 41,152] for the once-daily and twice-daily groups, respectively. In both dosing cohorts, there were no differences in follow-up SBP or DBP between the twice-daily and once-daily groups (**Table 1**). The distribution of achieved SBP and DBP categories was similar across dosing cohorts (**Fig 3**), and twice-daily dosing was not associated with greater odds of achieving any SBP or DBP category compared to once-daily dosing (**S3 Table**; all 95% CIs cross 1).

**Table 1:** Blood pressure outcomes among patients taking lisinopril daily or twice-daily for hypertension, by dosing cohort.

Outcome	Lisinopril						Losartan					
	20mg Cohort		Absolute mean difference (95% CI)	40mg Cohort		Absolute mean difference (95% CI)	50mg Cohort		Absolute mean difference (95% CI)	100mg Cohort		Absolute mean difference (95% CI)
	Daily (n = 6,156)	Twice-Daily (n = 230)		Daily (n = 4,258)	Twice-Daily (n = 549)		Daily (n = 2,734)	Twice-Daily (n = 186)		Daily (n = 2,864)	Twice-Daily (n = 258)	
SBP, mmHg	131.2 (130.8, 131.6)	131.9 (129.8, 134.0)	0.7 (-1.5, 2.8)	134.0 (133.5, 134.5)	133.4 (132.0, 134.8)	-0.6 (-2.1, 0.9)	133.7 (133.1, 134.2)	132.0 (129.7, 134.4)	-1.6 (-4.0, 0.7)	135.0 (134.3, 135.6)	133.1 (131.1, 135.2)	-1.8 (-4.0, 0.3)
DBP, mmHg	76.8 (76.6, 77.0)	76.4 (75.1, 77.7)	-0.4 (-1.7, 1.0)	77.4 (77.1, 77.7)	77.2 (76.3, 78.1)	-0.2 (-1.2, 0.7)	75.7 (75.4, 76.1)	75.8 (74.4, 77.3)	0.1 (-1.4, 1.6)	76.9 (76.6, 77.3)	76.1 (75.0, 77.2)	-1.1 (-2.5, 0.2)

Data are expressed as number (95% confidence interval) unless otherwise indicated.

Abbreviations: CI–confidence interval; DBP–diastolic blood pressure SBP–systolic blood pressure

Sensitivity analyses evaluating unadjusted and multivariable adjusted models similarly found no differences in SBP between once-daily and twice-daily groups in either dosing cohort (**S4 Table**). However, there were unadjusted differences in DBP between the twice-daily and once-daily groups of -3.0 mmHg (95% CI -4.4, -1.5) and -2.4 mmHg (95% CI -3.4, -1.3) in the 20mg and 40mg cohorts, respectively.

#### Losartan

In the losartan 50mg cohort, the average number of BP measurements in the 6 months prior to the index date was 4.2±3.2 and 3.1±4.7 in the once-daily and twice-daily groups, respectively, and the median time from index date to follow-up BP measurement was 71 days [IQR 36,152] and 74 days [IQR 37,128] for the once-daily and twice-daily groups, respectively. In the losartan 100mg cohort, the average number of BP measurements in the 6 months prior to the index date was 3.1±3.2 and 5.2±4.1 in the once-daily and twice-daily groups, respectively, and the median time from index date to follow-up BP measurement was 72 days [IQR 35,158] and 70 days [IQR 37,134] for the once-daily and twice-daily groups, respectively. Similar to lisinopril, in both losartan dosing cohorts, there were no differences in follow-up SBP or DBP between the twice-daily and once-daily groups (**Table 1**).

Similar to lisinopril, the distribution of achieved SBP and DBP categories was similar across dosing cohorts (**Fig 3**), but patients who were taking twice-daily had higher odds of achieving SBP <130 mmHg compared to once-daily in both dosing cohorts (**S5 Table**; 50mg OR 1.36, 95% CI 1.02, 1.82; 100mg OR 1.43, 95% CI 1.11, 1.86). Patients taking twice-daily had lower odds of achieving SBP 130–139 mmHg compared to once-daily in both dosing cohorts (50mg OR 0.54, 95% CI 0.38, 0.77; 100mg OR 0.74, 95% CI 0.56, 0.99). Additionally, patients who were taking losartan 25mg twice-daily had higher odds of achieving SBP 140–149 mmHg than those taking losartan 50mg once-daily (OR 1.61, 95% CI 1.09, 2.38). Finally, patients who were taking losartan 50mg twice-daily had lower odds of achieving DBP 90–99 mmHg than those taking losartan 100mg once-daily (OR 0.48, 95% CI 0.27, 0.86).

Significant differences in SBP and DBP observed in unadjusted models were rendered insignificant with multivariable adjustment (**S6 Table**), except for SBP in patients taking losartan 25mg twice-daily compared to losartan 50mg once-daily (absolute difference: -2.5 mmHg, 95% CI -4.8, -0.1).

### Adverse drug event outcomes

There were no differences in the incidence of ADEs in the lisinopril twice-daily group compared to the lisinopril once-daily group (**Table 2**), except that patients taking lisinopril 10mg twice-daily had greater odds of experiencing angioedema and hyperkalemia than patients taking lisinopril 20mg once-daily (angioedema OR 2.27, 95% CI 1.13, 4.58; hyperkalemia OR 2.54, 95% CI 1.31, 4.89). There were no differences in the incidence of ADEs in the losartan twice-daily group compared to the losartan once-daily group (**Table 3**).

**Table 2:** Adverse drug events among patients taking lisinopril once daily versus twice daily, by dosing cohort.

	20mg Cohort				40mg Cohort			
	Once-Daily (n = 6324)	Twice-Daily (n = 304)	Unweighted Odds Ratio (95% CI)	Weighted Odds Ratio (95% CI)	Once-Daily (n = 4276)	Twice-Daily (n = 529)	Unweighted Odds Ratio (95% CI)	Weighted Odds Ratio (95% CI)
AKI	210 (3.3)	19 (6.3)	1.94 (1.20, 3.15)	1.00 (0.52, 1.92)	168 (3.9)	27 (5.1)	1.32 (0.87, 2.00)	0.73 (0.43,1.22)
Angioedema	89 (1.4)	8 (2.6)	1.89 (0.91, 3.94)	2.27 (1.13, 4.58)	58 (1.4)	7 (1.3)	0.98 (0.44, 2.15)	1.21 (0.59, 2.48)
Hyperkalemia*	89 (1.4)	8 (2.6)	1.89 (0.91, 3.94)	2.54 (1.31, 4.89)	73 (1.7)	10 (1.9)	1.11 (0.57, 2.16)	1.21 (0.64, 2.29)

Data are expressed as unweighted number of events (unweighted percentage) unless otherwise indicated. Unweighted and inverse propensity score-weighted odds ratios are provided.

*Defined as follow-up serum potassium ≥5.2 mEq/L

Abbreviations: AKI–acute kidney injury; CI–confidence interval

**Table 3:** Adverse drug events among patients taking losartan once daily versus twice daily, by dosing cohort.

	50mg Cohort				100mg Cohort			
	Once-Daily (n = 2791)	Twice-Daily (n = 204)	Unweighted Odds Ratio (95% CI)	Weighted Odds Ratio (95% CI)	Once-Daily (n = 2963)	Twice-Daily (n = 348)	Unweighted Odds Ratio (95% CI)	Weighted Odds Ratio (95% CI)
AKI	120 (4.3)	8 (3.9)	0.91 (0.44, 1.89)	0.51 (0.20, 1.32)	106 (3.6)	15 (4.3)	1.21 (0.70, 2.11)	0.82 (0.43,1.56)
Angioedema	34 (1.2)	0 (0.0)	n/a	n/a	26 (0.9)	6 (1.7)	1.98 (0.81, 4.85)	1.26 (0.42, 3.75)
Hyperkalemia*	43 (1.5)	3 (1.5)	0.95 (0.29, 3.10)	0.56 (0.12, 2.60)	38 (1.3)	6 (1.7)	1.35 (0.57, 3.22)	1.01 (0.38, 2.65)

Data are expressed as unweighted number of events (unweighted percentage) unless otherwise indicated. Unweighted and inverse propensity score-weighted odds ratios are provided.

*Defined as follow-up serum potassium ≥5.2 mEq/L

Abbreviations: AKI–acute kidney injury; CI–confidence interval

## Discussion

In this retrospective study in an integrated healthcare delivery system, at least 7% of patients who were taking lisinopril or losartan were taking twice-daily dosing over seven years. Patients taking twice-daily lisinopril or losartan were more likely to be older with higher comorbidity and medication burdens than those taking once-daily. In IPTW-adjusted models, no statistically significant differences in SBP, DBP, or ADEs emerged between twice-daily and once-daily groups, except for an increased odds of experiencing angioedema and hyperkalemia with twice-daily dosing in the lower lisinopril dosing cohort. The increased risk of angioedema with ACEIs is consistent with data from the Food and Drug Administration (FDA) Mini-Sentinel Program [24], and likely occurred in the lower dosing cohort when patients are newly initiating an ACEI and more likely to experience a first-time ADE. The findings of this study have significant implications for understanding the degree to which clinicians deviate from traditional dosing strategies to treat high BP in real-world practice.

At the time of FDA approval for lisinopril (1987; Prinvil^®^, Merck&Co.) and losartan (1995; Cozaar^®^, Merck&Co.), the FDA evaluated antihypertensives using the trough-to-peak ratio (TPR) to determine if an agent should be dosed once-daily. If the agent’s TPR was at least 50%, it would be dosed once-daily (i.e., the effect at 24 hours after the administration of a dose must be at least 50% of the effect at the agent’s peak) [25–27]. Lisinopril’s TPR ranges from 30–70%, and losartan’s TPR is 70% [28, 29]. The TPR relies on assessment of BP at only two time points and largely ignores the impacts of circadian rhythm or other activities that may be better assessed using ambulatory BP monitoring. In the clinical setting, other patient-specific factors such as medication adherence may also impact the decision to use nontraditional dosing frequencies. As such, clinicians often employ a personalized approach to drug dosing by modifying the dosing frequency according to patient response, similar to published *n*-of-1 trials [30, 31]; this strategy applied to altering dosing frequency has yet to be formally investigated as an effective means to improve BP control.

The current analysis provides evidence of the extent to which clinicians deviate from manufacturer-labeled recommendations for two commonly-used medications for high BP. Of 11,210 and 6,051 patients who met all inclusion and exclusion criteria for lisinopril and losartan, 795 (7.1%) and 453 (7.5%) were taking twice-daily, respectively. Assuming that this prevalence mirrors general clinical practice and that 30 million Americans are prescribed lisinopril or losartan [32], it is possible that approximately 2.1 million Americans are taking twice-daily lisinopril or losartan. More broadly, the prevalence of nontraditional dosing strategies for antihypertensive medications has not been investigated, and further, it is unknown whether nontraditional dosing strategies for hypertension treatment impact BP and clinical outcomes. Patients who are taking nontraditional antihypertensive medication regimens may serve as target populations for interventions to improve metrics and goals for BP control.

Our findings support data generated from randomized trials and prospective cohort studies that have demonstrated no statistically significant differences in BP reduction or tolerability between once-daily and twice-daily dosing strategies of other ACEIs (ramipril, enalapril, trandolapril) and ARBs (losartan, eprosartan, irbesartan, olmesartan, valsartan, candesartan) [33–41]. However, these studies lack generalizability to current practice by including or evaluating patients based on DBP values [33, 34, 36–38, 41], excluding key chronic disease populations like heart failure or chronic kidney disease [33–37, 41], or comparing dosing against placebo [36, 38, 41]. It is possible that pharmacokinetic data demonstrate greater BP reduction with twice-daily dosing in adults studied in controlled environments. However, the effectiveness and safety findings described in the current real-world study combined with adherence data supporting once-daily regimens [42–45] suggest that less-complex regimens should be favored for hypertension treatment. Applying evidence-based interventions that aim to reduce pill burden or reduce medication regimen complexity (e.g., deprescribing, using fixed-dose/single-pill combinations) may improve BP control, especially among those with resistant hypertension.

The current study findings oppose our previous work [9] for several reasons. Thousands of patients met the inclusion criteria for all four dosing cohorts, compared to 90 patients for one dosing cohort previously. The previous study excluded patients who had a change in medications that might impact BP between baseline and follow-up measurements; whereas, the current study included concomitant BP-affecting medications in the propensity score model. The two studies have different analytic models, as the current study used an extensive set of covariates to generate a propensity score, which was used in IPTW-adjusted models; however, small differences (<5 mmHg) in BP were only significant in unadjusted models for losartan. Unlike our previous study, given the large sample size, we were not able to review each medical record to confirm dosing regimens or the dosing regimen used at the time of the follow-up BP measurement. Finally, the average baseline BP in the lisinopril groups of the current study were lower than the baseline BP measurements in our previous study, and we currently included patients regardless of baseline BP control. Therefore, it is possible that patients included in the current study may have had controlled BP and underwent a change in dosing for other reasons, biasing the effect toward the null.

The current study is also limited by the retrospective design in one health system. Due to the large sample size created using administrative queries, we were not able to review all records to confirm indication for lisinopril or losartan, purpose of dose increase, or adverse effects not detectable with administrative queries (e.g., dry cough for lisinopril). It is possible that patient-specific factors such as morning BP measurements or poor response to once-daily dosing could have influenced clinicians’ decisions to change the dosing frequency from once- to twice-daily, and therefore, could have impacted our findings. Further, we did not analyze adherence outcomes, but literature well-describes decrements in adherence with multiple-daily dosing [42–45]. Clinical decision-making by providers is highly complex, and both patient- and provider-specific factors may have influenced the decision to use twice-daily dosing over once-daily dosing. Factors such as adherence, BP measurements throughout the day, previous adverse responses to medication doses, use of non-antihypertensive medications, diet, stress, and medication adherence, among other factors, could impact a clinician’s dosing decision. We accounted for several measured covariates with a propensity score model, however, it is possible that such complex or unmeasured factors could have impacted our findings. The high level of missingness for prescriber specialty, although it did not impact estimation of the propensity score, could impact findings because a clinician may manage medications differently depending on his or her training background. The BP measurements used in this study are subject to constraints of real-world BP measurement practices over 10 years. While it is impossible to completely adjust for these constraints, we attempted to mitigate potential confounding by including time to first BP measurement after the index date in the analysis, excluding BP measurements from inpatient or acute settings, averaging BP measurements taken on the same date, and removing non-plausible BP measurements (e.g., SBP 300 mmHg). Future improvements in BP measurement techniques or measurements observed with ambulatory BP monitoring (e.g., mean 24-hour BP or morning BP values) could impact our findings. Finally, the low number of adverse events may have limited our ability to detect significant differences between once- and twice-daily dosing, and our findings should be interpreted within the context of the wide confidence intervals.

For these two commonly-prescribed medications, over 7% of patients were on this nontraditional dosing strategy. These findings should be validated in other health systems and considered by clinicians when implementing creative methods of maximizing antihypertensive medication regimens. Future research may highlight the prevalence of nontraditional antihypertensive dosing regimens and the improvements in clinical outcomes associated with reducing pill burden or medication complexity among patients taking these regimens.

## Supporting information

S1 Table Baseline characteristics of lisinopril daily and twice-daily groups before IPTW, by dose cohort. (PDF)

S2 Table Baseline characteristics of losartan daily and twice-daily groups before IPTW, by dose cohort. (PDF)

S3 Table Odds ratios for achieving each systolic and diastolic blood pressure category among patients taking lisinopril once-daily or twice-daily for hypertension, by dosing cohort. (PDF)

S4 Table Blood pressure outcomes among patients taking lisinopril daily or twice-daily for hypertension, by dosing cohort and analytic model. (PDF)

S5 Table Odds ratios for achieving each systolic and diastolic blood pressure category among patients taking losartan once-daily or twice-daily for hypertension, by dosing cohort. (PDF)

S6 Table Blood pressure outcomes among patients taking losartan daily or twice-daily for hypertension, by dosing cohort and analytic model. (PDF)
