# Supplementary material for: Twice-daily versus once-daily lisinopril and losartan for hypertension: Real-world effectiveness and safety
Source: PLoS One. 2020 Dec 3;15(12):e0243371. doi: 10.1371/journal.pone.0243371 (PMC7714357; doi:10.1371/journal.pone.0243371)
Supplement: S1 Table — (PDF) [file pone.0243371.s001.pdf]

S1 Table: Baseline characteristics of lisinopril daily and twice-daily groups before IPTW, by dose cohort						
Characteristic	20 mg Cohort		p-value	40 mg Cohort		p-value
	Daily (n=6156)	Twice-Daily (n=235)		Daily (n=4249)	Twice-Daily (n=560)	
Age, years	63.0±13.1	70.6±12.4	<0.001	63.8±12.5	68.5±12.8	<0.001
Female sex	2846 (46.2)	111 (47.2)	0.05	1900 (44.6)	263 (47.0)	0.29
Race						
White	4502 (73.1)	192 (81.7)	0.004	3065 (72.0)	417 (74.5)	0.072
Other	956 (15.5)	26 (11.1)	0.06	686 (16.1)	79 (14.1)	0.23
Undeclared/Unknown	698 (11.3)	17 (7.2)	0.06	508 (11.9)	64 (11.4)	0.73
Hispanic Ethnicity						
Hispanic	867 (14.1)	21 (8.9)	0.03	602 (14.1)	69 (12.3)	0.24
Non-Hispanic	5123 (83.2)	213 (90.6)	0.003	3560 (83.6)	469 (83.8)	0.92
Undeclared/Unknown	166 (2.7)	1 (0.4)	0.03	97 (2.3)	22 (3.9)	0.02
Tobacco use						
Current	770 (12.5)	14 (6.0)	0.002	567 (13.3)	53 (9.5)	0.01
Former	2319 (37.7)	90 (38.3)	0.85	1592 (37.4)	246 (43.9)	0.003
Never	3062 (49.7)	131 (55.7)	0.07	2097 (49.2)	261 (46.6)	0.24
Unknown/Missing	5 (0.1)	0 (0.0)	0.99	3 (0.1)	0 (0.0)	0.99
Median income, US Dollars ($)	$81,766 ±$30,407	$82,780 ±$30,966	0.97	$80,327± $30,061	$83,414± $31,545	0.008
Some college education, %	90.0±10.0	90.9±8.9	0.47	89.7±10.1	90.9±9.7	0.001
BMI kg/m^2	30.6±6.7	28.4±5.9	<0.001	31.2±7.1	29.9±6.7	<0.001
Underweight	52 (0.8)	5 (2.1)	0.06	23 (0.5)	4 (0.7)	0.19
Normal weight	1042 (17.0)	58 (24.7)	0.002	654 (15.4)	103 (18.4)	0.06
Overweight	1910 (31.0)	74 (31.5)	0.88	1277 (30.0)	186 (33.2)	0.12
Obese	2693 (43.6)	77 (32.8)	<0.001	2010 (47.2)	213 (38.0)	<0.001
Missing	459 (7.5)	21 (8.9)	0.38	295 (6.9)	54 (9.6)	0.02
Charlson Comorbidity Index	3.2±2.4	4.4±2.7	<0.001	3.4±2.4	4.3±2.7	<0.001
Comorbidities						
Chronic pulmonary disease	1204 (19.6)	51 (21.7)	0.42	810 (19.0)	139 (24.8)	0.001
Congestive heart failure	468 (7.6)	47 (20.0)	<0.001	298 (7.0)	80 (14.3)	<0.001
Depression	976 (15.9)	52 (22.1)	0.01	724 (17.0)	126 (22.5)	0.001
Diabetes	1567 (25.5)	66 (28.1)	0.36	1291 (30.3)	163 (29.1)	0.56
Chronic kidney disease	1202 (19.5)	65 (27.7)	0.002	1005 (23.6)	192 (34.3)	<0.001
SBP, mm Hg	139.4±17.7	137.1±21.2	0.06	142.3±18.2	140.2±20.2	0.01
DBP, mm Hg	81.0±12.5	75.9±13.2	<0.001	81.9±12.6	78.9±12.9	<0.001
Serum creatinine, mg/dL	1.0±0.5	1.1±0.7	0.005	1.0±0.5	1.2±1.1	0.05
Mean eGFR, mL/min/1.73m^2	70.8±22.7	64.5±22.0	<0.001	69.2±22.7	62.8±23.0	<0.001
<30 mL/min/1.73m^2	75 (1.2)	6 (2.6)	0.12	69 (1.6)	23 (4.1)	<0.001
30-59 mL/min/1.73m^2	1212 (19.7)	70 (29.8)	<0.001	958 (22.5)	161 (28.8)	0.001
≥60 mL/min/1.73m^2	2863 (46.5)	98 (41.7)	0.15	1980 (46.5)	231 (41.3)	0.02
Missing	2006 (32.6)	61 (26.0)	0.03	1252 (29.4)	145 (25.9)	0.09
Serum potassium, mEq/L	4.2±0.4	4.2±0.4	0.56	4.2±0.4	4.2±0.4	0.34
Urinary ACR, mg/g	245.8±706.5	333.7±1070.0	0.55	285.9±727.4	474.3±1140.3	0.29
Concomitant medications						
Anti-anginal	106 (1.7)	11 (4.7)	<0.001	68 (1.6)	25 (4.5)	<0.001
Beta-blocker	1612 (26.2)	110 (46.8)	<0.001	1341 (31.5)	276 (49.3)	<0.001

Calcium channel blocker	415 (6.7)	38 (16.2)	<0.001	615 (14.4)	108 (19.3)	0.003
Diuretic	788 (12.8)	46 (19.6)	0.003	636 (14.9)	147 (26.3)	<0.001
Glucocorticosteroid	194 (3.2)	9 (3.8)	0.57	138 (3.2)	27 (4.8)	0.05
Immunosuppressants	26 (0.4)	3 (1.9)	0.09	12 (0.3)	5 (0.9)	0.04
MAOI	1 (0.02)	0 (0.0)	0.99	0 (0.0)	(0.0)	0.99
Mineralocorticoids	2 (0.03)	1 (0.4)	0.11	2 (0.05)	1 (0.2)	0.31
Nicotine replacement	6 (0.1)	0 (0.0)	0.99	4 (0.1)	0 (0.0)	0.99
NSAIDs	349 (5.7)	9 (3.8)	0.31	247 (5.8)	24 (4.3)	0.13
SNRI	136 (2.2)	6 (2.6)	0.65	122 (2.9)	14 (2.9)	0.79

Data are expressed as mean ± standard deviation or number (percentage) unless otherwise indicated.
Abbreviations: ACR = albumin-to-creatinine ratio; BMI = body mass index; DBP = diastolic blood pressure; IPTW = inverse probability of treatment weighting; SBP = systolic blood pressure
