# Supplementary material for: Twice-daily versus once-daily lisinopril and losartan for hypertension: Real-world effectiveness and safety
Source: PLoS One. 2020 Dec 3;15(12):e0243371. doi: 10.1371/journal.pone.0243371 (PMC7714357; doi:10.1371/journal.pone.0243371)
Supplement: S2 Table — (PDF) [file pone.0243371.s002.pdf]

S2 Table: Baseline characteristics of losartan daily and twice-daily groups before IPTW, by dose cohort.						
Characteristic	50 mg Cohort		p-value	100 mg Cohort		p-value
	Daily (n=2,734)	Twice-Daily (n=190)		Daily (n=2,864)	Twice-Daily (n=263)	
Age, years	67.1±11.9	69.3±12.1	0.01	66.0±11.7	70.4±11.2	<0.001
Female sex	1653 (60.2)	120 (63.2)	0.46	1647 (57.5)	159 (60.5)	0.35
Race						
White	2014 (73.4)	157 (82.6)	0.006	2017 (70.4)	208 (79.1)	0.003
Other	464 (17.0)	19 (10.0)	0.01	550 (19.2)	40 (15.2)	0.11
Undeclared/Unknown	256 (9.4)	14 (7.4)	0.07	297 (10.4)	15 (5.7)	0.01
Hispanic Ethnicity						
Hispanic	341 (12.5)	11 (5.8)	0.005	396 (13.8)	26 (9.9)	0.07
Non-Hispanic	2347 (85.8)	172 (90.5)	0.07	2421 (84.5)	232 (88.2)	0.11
Undeclared/Unknown	46 (1.7)	7 (3.7)	0.08	47 (1.6)	5 (1.9)	0.62
Tobacco use						
Current	192 (7.0)	14 (7.4)	0.88	219 (7.7)	10 (3.8)	0.02
Former	1091 (39.9)	85 (44.7)	0.19	1101 (38.4)	116 (44.1)	0.07
Never	1451 (53.1)	91 (47.9)	0.17	1543 (53.9)	137 (52.1)	0.58
Unknown/Missing	0 (0.0)	0 (0.0)	0.99	1 (0.03)	0 (0.0)	0.99
Median income, US Dollars ($)	$82,471± $30,774	$89,266± $33,545	0.005	$82,101± $30,971	$87,449± $34,069	0.03
Some college education, %	90.4±9.9	92.3±8.6	0.001	90.2±9.8	91.0±9.7	0.008
BMI kg/m^2	30.9±6.8	28.5±5.3	<0.001	31.4±6.9	29.3±6.1	<0.001
Underweight	14 (0.5)	3 (1.6)	0.09	14 (0.5)	5 (1.9)	0.02
Normal weight	453 (16.6)	44 (23.2)	0.02	414 (14.5)	56 (21.3)	0.003
Overweight	858 (31.4)	70 (36.8)	0.12	867 (30.3)	90 (34.2)	0.18
Obese	1276 (46.7)	63 (33.2)	<0.001	1412 (49.3)	100 (38.0)	<0.001
Missing	133 (4.9)	10 (5.3)	0.73	157 (5.5)	12 (4.6)	0.67
Charlson Comorbidity Index	4.1±2.6	5.1±3.1	<0.001	3.8±2.5	4.9±3.0	<0.001
Comorbidities						
Chronic pulmonary disease	753 (27.5)	65 (34.2)	0.05	708 (24.7)	86 (32.7)	0.004
Congestive heart failure	344 (12.6)	54 (28.4)	<0.001	263 (9.2)	59 (22.4)	<0.001
Depression	537 (19.6)	42 (22.1)	0.41	577 (20.2)	61 (23.2)	0.24
Diabetes	943 (34.5)	61 (32.1)	0.50	940 (32.8)	85 (32.3)	0.87
Chronic kidney disease	853 (31.2)	70 (36.8)	0.11	812 (28.4)	91 (34.6)	0.03
SBP, mm Hg	139.5±18.0	135.6±19.4	0.004	143.4±18.5	139.5±20.5	0.001
DBP, mm Hg	78.7±12.1	74.4±10.8	<0.001	81.0±12.7	77.3±12.5	<0.001
Serum creatinine, mg/dL	1.0±0.5	1.1±0.8	0.60	1.0±0.6	1.0±0.4	0.30
Mean eGFR, mL/min/1.73m^2	66.3±22.7	64.8±23.2	0.44	67.3±22.4	64.1±21.5	0.05
<30 mL/min/1.73m2	75 (2.7)	6 (3.2)	0.65	62 (2.2)	5 (1.9)	0.99
30-59 mL/min/1.73m2	728 (26.6)	56 (29.5)	0.39	756 (26.4)	86 (32.7)	0.03
≥60 mL/min/1.73m2	1302 (47.6)	81 (42.6)	0.18	1396 (48.7)	113 (43.0)	0.07
Missing	629 (23.0)	47 (24.7)	0.58	650 (22.7)	59 (22.4)	0.92
Serum potassium, mEq/L	4.2±0.4	4.2±0.4	0.61	4.1±0.4	4.2±0.4	0.23
Urinary ACR, mg/g	277.3±745.4	570.5±1245.5	0.29	313.7±822.4	331.8±931.7	0.32
Concomitant medications						
Anti-anginal	82 (3.0)	17 (9.0)	<0.001	59 (2.1)	18 (6.8)	<0.001
Beta-blocker	958 (35.0)	90 (47.4)	<0.001	963 (33.6)	132 (50.2)	<0.001
Calcium channel blocker	404 (14.8)	38 (20.0)	0.05	563 (19.7)	49 (18.6)	0.69

Diuretic	747 (27.3)	67 (35.3)	0.02	745 (26.0)	93 (35.4)	0.001
Glucocorticosteroid	91 (3.3)	6 (3.2)	0.99	104 (3.6)	15 (5.7)	0.09
Immunosuppressants	14 (0.5)	4 (2.1)	0.03	13 (0.5)	4 (1.5)	0.05
MAOI	0 (0.0)	0 (0.0)	0.99	0 (0.0)	0 (0.0)	0.99
Mineralocorticoids	0 (0.0)	0 (0.0)	0.99	2 (0.1)	0 (0.0)	0.99
Nicotine replacement	0 (0.0)	0 (0.0)	0.99	1 (0.03)	0 (0.0)	0.99
NSAIDs	129 (4.7)	6 (3.2)	0.47	154 (5.4)	15 (5.7)	0.78
SNRI	105 (3.8)	6 (3.2)	0.63	83 (2.9)	12 (4.6)	0.13

Data are expressed as mean ± standard deviation or number (percentage) unless otherwise indicated.
Abbreviations: ACR = albumin-to-creatinine ratio; BMI = body mass index; DBP = diastolic blood pressure; IPTW = inverse probability of treatment weighting; SBP = systolic blood pressure
