# Supplementary material for: Twice-daily versus once-daily lisinopril and losartan for hypertension: Real-world effectiveness and safety
Source: PLoS One. 2020 Dec 3;15(12):e0243371. doi: 10.1371/journal.pone.0243371 (PMC7714357; doi:10.1371/journal.pone.0243371)
Supplement: S3 Table — (PDF) [file pone.0243371.s003.pdf]

S3 Table: Odds ratios for achieving each systolic and diastolic blood pressure category among patients taking lisinopril once-daily or twice-daily for hypertension, by dosing cohort.

Outcome	20 mg Cohort Daily (n=6,156)	20 mg Cohort Twice-Daily (n=230)	Odds Ratio (95% CI)	40 mg Cohort Daily (n=4,258)	40 mg Cohort Twice-Daily (n=549)	Odds Ratio (95% CI)
**SBP**						
SBP <130 mm Hg	2793 (45.4)	107 (46.5)	0.97 (0.74, 1.27)	1660 (39.0)	236 (43.0)	1.10 (0.91, 1.32)
SBP 130-139 mm Hg	1890 (30.7)	74 (32.2)	1.20 (0.91, 1.60)	1343 (31.5)	158 (28.8)	0.93 (0.77, 1.13)
SBP 140-149 mm Hg	645 (10.5)	20 (8.7)	0.74 (0.45, 1.22)	496 (11.7)	59 (10.8)	1.00 (0.76, 1.32)
SBP ≥150 mm Hg	828 (13.5)	29 (12.6)	0.94 (0.63, 1.41)	759 (17.8)	96 (17.5)	0.95 (0.75, 1.21)
**DBP**						
DBP <80 mm Hg	3408 (55.4)	154 (67.0)	1.14 (0.86, 1.49)	2255 (53.0)	340 (61.9)	1.06 (0.89, 1.27)
DBP 80-89 mm Hg	2052 (33.3)	58 (25.2)	0.93 (0.70, 1.25)	1411 (33.1)	153 (27.9)	0.95 (0.78, 1.15)
DBP 90-99 mmHg	504 (8.2)	14 (6.1)	0.97 (0.58, 1.60)	411 (9.7)	39 (7.1)	0.92 (0.67, 1.26)
DBP ≥100 mm Hg	192 (3.1)	4 (1.7)	0.58 (0.21, 1.59)	181 (4.3)	17 (3.1)	1.10 (0.71, 1.70)

Data are expressed as number (percentage) unless otherwise indicated.
Abbreviations: CI = confidence interval; DBP = diastolic blood pressure SBP = systolic blood pressure
