# Supplementary material for: Twice-daily versus once-daily lisinopril and losartan for hypertension: Real-world effectiveness and safety
Source: PLoS One. 2020 Dec 3;15(12):e0243371. doi: 10.1371/journal.pone.0243371 (PMC7714357; doi:10.1371/journal.pone.0243371)
Supplement: S4 Table — (PDF) [file pone.0243371.s004.pdf]

S4 Table: Blood pressure outcomes among patients taking lisinopril daily or twice-daily for hypertension, by dosing cohort and analytic model.

**Outcome**	**20 mg Cohort** Daily (n=6,156)	**20 mg Cohort** Twice-Daily (n=230)	**Absolute difference (95% CI)**	**40 mg Cohort** Daily (n=4,258)	**40 mg Cohort** Twice-Daily (n=549)	**Absolute difference (95% CI)**
SBP at follow-up, mm Hg						
Unadjusted	131.2 (130.8, 131.7)	131.4 (129.1, 133.7)	0.2 (-2.1, 2.4)	134.1 (133.5, 134.6)	132.8 (131.2, 134.3)	-1.3 (-2.9, 0.3)
Multivariable-adjusted*	131.2 (130.8, 131.6)	131.6 (129.5, 133.7)	0.4 (-1.8, 2.5)	134.0 (133.5, 134.5)	133.1 (131.7, 134.5)	-0.9 (-2.5, 0.6)
IPTW	131.2 (130.8, 131.6)	131.9 (129.8, 134.0)	0.7 (-1.5, 2.8)	134.0 (133.5, 134.5)	133.4 (132.0, 134.8)	-0.6 (-2.1, 0.9)
DBP at follow-up, mm Hg						
Unadjusted	76.9 (76.6, 77.2)	73.9 (72.5, 75.4)	-3.0 (-4.4, -1.5)	77.7 (77.3, 78.0)	75.3 (74.3, 76.3)	-2.4 (-3.4, -1.3)
Multivariable-adjusted*	76.8 (76.6, 77.0)	77.0 (75.7, 78.3)	0.2 (-1.1, 1.5)	77.4 (77.1, 77.7)	77.1 (76.3, 78.0)	-0.3 (-1.2, 0.7)
IPTW	76.8 (76.6, 77.0)	76.4 (75.1, 77.7)	-0.4 (-1.7, 1.0)	77.4 (77.1, 77.7)	77.2 (76.3, 78.1)	-0.2 (-1.2, 0.7)

Data are expressed as mean (95% confidence intervals) unless otherwise indicated.
*****Adjusted for gender, age, race, ethnicity, tobacco use, BMI, number of BP measurements before the index date, CCI, chronic pulmonary disease, congestive heart failure, depression, diabetes, chronic kidney disease, eGFR, albuminuria, other medications that affect BP, and provider specialty.
Abbreviations: CI = confidence interval; DBP = diastolic blood pressure SBP = systolic blood pressure
