# Supplementary material for: Twice-daily versus once-daily lisinopril and losartan for hypertension: Real-world effectiveness and safety
Source: PLoS One. 2020 Dec 3;15(12):e0243371. doi: 10.1371/journal.pone.0243371 (PMC7714357; doi:10.1371/journal.pone.0243371)
Supplement: S5 Table — (PDF) [file pone.0243371.s005.pdf]

S5 Table: Odds ratios for achieving each systolic and diastolic blood pressure category among patients taking losartan once-daily or twice-daily for hypertension, by dosing cohort.

Outcome	50 mg Cohort		Odds Ratio (95% CI)	100 mg Cohort		Odds Ratio (95% CI)
	Daily (n=2,734)	Twice-Daily (n=186)		Daily (n=2,864)	Twice-Daily (n=258)	
**SBP**						
SBP <130 mm Hg	1094 (39.2)	105 (51.5)	1.36 (1.02, 1.82)	1015 (35.4)	116 (45.0)	1.43 (1.11, 1.86)
SBP 130-139 mm Hg	930 (33.3)	48 (23.5)	0.54 (0.38, 0.77)	980 (34.2)	71 (27.5)	0.74 (0.56, 0.99)
SBP 140-149 mm Hg	318 (11.4)	26 (12.8)	1.61 (1.09, 2.38)	359 (12.5)	28 (10.9)	0.89 (0.59, 1.33)
SBP ≥150 mm Hg	449 (16.1)	25 (12.3)	0.91 (0.61, 1.37)	510 (17.8)	43 (16.7)	0.93 (0.66, 1.31)
**DBP**						
DBP <80 mm Hg	1656 (59.3)	141 (69.1)	1.01 (0.75, 1.35)	1559 (54.4)	169 (65.5)	1.26 (0.97, 1.63)
DBP 80-89 mm Hg	834 (29.9)	52 (25.5)	1.13 (0.83, 1.54)	917 (32.0)	71 (27.5)	1.00 (0.77, 1.33)
DBP 90-99 mmHg	213 (7.6)	9 (4.4)	0.96 (0.55, 1.68)	286 (10.0)	13 (5.0)	0.48 (0.27, 0.86)
DBP ≥100 mm Hg	88 (3.2)	2 (1.0)	0.22 (0.04, 1.20)	102 (3.6)	5 (1.9)	0.74 (0.33, 1.65)

Data are expressed as number (percentage) unless otherwise indicated.
Abbreviations: CI = confidence interval; DBP = diastolic blood pressure SBP = systolic blood pressure
