# Supplementary material for: Twice-daily versus once-daily lisinopril and losartan for hypertension: Real-world effectiveness and safety
Source: PLoS One. 2020 Dec 3;15(12):e0243371. doi: 10.1371/journal.pone.0243371 (PMC7714357; doi:10.1371/journal.pone.0243371)
Supplement: S6 Table — (PDF) [file pone.0243371.s006.pdf]

S6 Table: Blood pressure outcomes among patients taking losartan daily or twice-daily for hypertension, by dosing cohort and analytic model.

Outcome	50 mg Cohort Daily (n=2,734)	50 mg Cohort Twice-Daily (n=186)	Absolute difference (95% CI)	100 mg Cohort Daily (n=2,864)	100 mg Cohort Twice-Daily (n=258)	Absolute difference (95% CI)
SBP at follow-up, mm Hg						
Unadjusted	133.8 (133.1, 134.4)	129.4 (126.9, 132.0)	-4.3 (-6.8, -1.8)	135.1 (134.3, 135.7)	132.2 (129.9, 134.4)	-2.9 (-5.1, -0.7)
Multivariable-adjusted*	133.6 (133.1, 134.2)	131.2 (128.9, 133.5)	-2.5 (-4.8, -0.1)	135.0 (134.4, 135.6)	133.3 (131.2, 135.3)	-1.7 (-3.9, 0.4)
IPTW	133.7 (133.1, 134.2)	132.0 (129.7, 134.4)	-1.6 (-4.0, 0.7)	135.0 (134.3, 135.6)	133.1 (131.1, 135.2)	-1.8 (-4.0, 0.3)
DBP at follow-up, mm Hg						
Unadjusted	75.9 (75.4, 76.3)	73.3 (71.8, 74.8)	-2.6 (-4.2, -0.9)	77.3 (76.9, 77.7)	74.2 (72.8, 75.6)	-3.2 (-4.6, -1.7)
Multivariable-adjusted*	75.7 (75.4, 76.1)	75.5 (74.1, 76.9)	-0.2 (-1.7, 1.2)	77.1 (76.8, 77.5)	76.4 (75.2, 77.6)	-0.7 (-2.0, 0.6)
IPTW	75.7 (75.4, 76.1)	75.8 (74.4, 77.3)	0.1 (-1.4, 1.6)	76.9 (76.6, 77.3)	76.1 (75.0, 77.2)	-1.1 (-2.5, 0.2)

Data are expressed as mean (95% confidence intervals) unless otherwise indicated.
*Adjusted for gender, age, race, ethnicity, tobacco use, BMI, number of BP measurements before the index date, CCI, chronic pulmonary disease, congestive heart failure, depression, diabetes, chronic kidney disease, eGFR, albuminuria, other medications that affect BP, and provider specialty.
Abbreviations: CI = confidence interval; DBP = diastolic blood pressure SBP = systolic blood pressure
